# Robot-assisted treatment of unstable pelvic fractures with a percutaneous iliac lumbar double rod fixation combined with a percutaneous pelvic anterior ring INFIX fixation

**DOI:** 10.1007/s00264-020-04522-z

**Published:** 2020-04-21

**Authors:** Wei Du, Tao Sun, Yan Ding, Chuanqiang Jiang, Wenqing Qu, Shudong Zhang

**Affiliations:** 1 Department of Spine, Yantaishan Hospital, Yantai, 264000 Shandong China; 2 Department of Traumatology, Yantaishan Hospital, Yantai, 264000 Shandong China; 3 Department of Joint, Yantaishan Hospital, No. 91, Jiefang Road, Yantai, 264000 Shandong China

**Keywords:** Unstable pelvic fracture, Robot-assisted surgery, Minimally invasive surgery, Iliac lumbar fixation, Internal fixator

## Abstract

**Objective:**

To investigate the clinical effect of robot-assisted treatment of unstable pelvic fractures through a percutaneous iliac lumbar double rod fixation combined with a percutaneous pelvic anterior ring INFIX (internal fixator) fixation.

**Methods:**

This was a retrospective analysis of 17 cases of unstable anterior and posterior pelvic ring fractures treated between April 2016 and October 2018 by the third Ti-robot system produced in China. The posterior ring was supported with an iliac lumbar double rod fixation and the anterior ring with an INFIX fixation. Operation time and peri-operative bleeding were recorded. The reduction of pelvic fracture displacement was evaluated by Matta score, the post-operative results were evaluated according to Majeed score, and the complications were recorded.

**Results:**

Twelve males and five females, aged 21–71 years (mean 40.1 ± 3.8 years) were followed up for three to 12 months, (median 6.7 months). Tile typing showed seven B1 type, two B2 type, and eight C1 type cases. Operation time was 90–160 minutes (mean 112.9 ± 16.8 minutes), bleeding was 80–150 mL (mean 105.9 ± 20.6 mL). X-ray three to five  days after operation was evaluated by Matta score as excellent in 15 and good in two cases. Majeed score at last follow-up was 85–98 points, excellent in 17 cases. Two cases of lower extremity deep vein thrombosis received an inferior vena cava filter. The filters were removed after two  weeks. One case showed incision fat liquefaction healing and the wound healed three  weeks after surgery.

**Conclusion:**

Orthopedic robot-assisted treatment of unstable pelvic fractures by a percutaneous iliac lumbar double rod fixation and a percutaneous pelvic anterior ring INFIX fixator was minimally invasive and feasible. A prospective study is needed.

## Introduction

Although fractures and injuries of the pelvic ring account for only 2–8% of all fractures [1], an unstable pelvic fracture is associated with a high risk of mortality [2]. Unstable pelvic ring fractures require both anterior and posterior pelvic ring fixation [1, 3]. Traditional methods include external fixation, anterior and posterior open reduction plate internal fixation, and minimally invasive percutaneous screw fixation [4]. Open reduction and internal fixation of pelvic fractures can result in a large amount of tissue damage, increased bleeding, and nerve and vascular injury with complications, such as wound disunion and infection [4]. Minimally invasive closed reduction or percutaneous fixation aims to reduce these problems while still providing reliable fixation [5–8]. Recently, computer navigation and robot-assisted minimally invasive internal fixation have assisted orthopaedic surgery [9]. These methods provide accurate localization, low trauma, short operation time, and low radiation dose.

Traditional posterior ring fixator methods include external fixation, open iliac lumbar fixation, plate fixation and minimally invasive percutaneous sacroiliac screw fixation [7]. Iliac screw reconstruction of the lumbosacral junction has superior biomechanical characteristics compared to other internal fixation methods, with favourable resistance to stress failure [10]. Double iliac screws and double iliac screws with two rods are superior to single iliac screws for pelvic stability [11–14]. A double rod fixation provides stronger stability for an iliac lumbar fixation, while percutaneous minimally invasive surgery with single iliac screws for degenerative lumbar deformity significantly reduces complications [15]. This technique was improved and developed in patients with lumbosacral metastatic tumours into a minimally invasive double iliac screw-double rod technique, with good spinal stability [16, 17]. Therefore, using robotic navigation, we further improved this by linking the L4 pedicle screw end with the cephalic iliac screw end and linking the L5 pedicle screw end with the caudal iliac screw end as a percutaneous two-point puncture rod is easier.

For anterior ring injury, traditional fixation methods include external fixation, open reduction plate fixation, X-ray guidance for ramus screw fixation, and open Stoppa approach surgery [18]. The INFIX, a new minimally invasive method for the treatment of unstable pelvic fractures, was described in 2011 with little trauma and little impact on patients’ daily activities [19]. It is especially suitable for obese patients. However, this approach requires a small incision and the pedicle screw is inserted by hand between the internal and external cortex of the iliac crest at the anterior inferior iliac spine. In this position, the Medullary cavity between the iliac internal and external cortex is narrowed. Blunt subcutaneous separation of the anterior inferior iliac spine in obese patients could lead to injury of the lateral femoral cutaneous nerve. Therefore, robot assistance is helpful.

The Ti-robot is the most advanced orthopaedic robot system independently developed in China. Currently, it is used in spinal and traumatic internal fixation surgery with puncture accuracy of 0.6–0.8 mm. The purpose of this study is to describe the application of robot-assisted minimally invasive techniques for the treatment of pelvic fractures. Including consideration of treatment rationality, surgical indications, surgical techniques, and clinical results.

## Materials and methods

### Patients

This was a retrospective study of patients who received robot-assisted treatment of unstable pelvic fractures admitted between April 2016 and October 2018 in the department of traumatic orthopaedics, Yantaishan Hospital, Yantai City. The inclusion criteria were as follows: (1) closed pelvic anterior and posterior ring injuries; (2) unilateral posterior ring instability, combined with anterior ring instability pelvic fractures (tile typing: B1, B2, C1); (3) without severe internal organ injury; (4) without obvious fracture displacement, displacement or reduction by pre-operative or intra-operative traction. The exclusion criteria were as follows: (1) patients with severe visceral organ injury such as bladder rupture, urethral rupture, or intestinal rupture that required emergency laparotomy and (2) significant wound contamination.

After admission, patients’ vital signs were detected, venous access was established, urine catheterization was conducted, and circulating blood volume (crystal fluid, colloidal fluid, plasma, and red blood cells) was supplemented. Patients with circulatory instability were treated in the intensive care unit (ICU) with external pelvic fixation. The sacroiliac joint was separated, and bone traction was performed on the lower limbs. All patients underwent X-rays, computed tomography (CT) scan, and 3D reconstruction. Seventy-two hours after injury, X-rays were used to evaluate the effect of reduction with haemodynamically stable patients. Surgery was performed after five to 14 days. All patients accepted iliac lumbar fixation with cannulated pedicle screw and anterior ring INFIX fixation. All patients signed the informed consent before surgery. Robot surgery, posterior iliac waist fixation, and anterior ring INFIX fixation and the research were all approved by the Ethics Committee of Yantaishan Hospital in Yantai city.

### Surgical equipment

The China TINAVI Medical Co., Ltd. produced the third generation Ti-robot system, which includes robots, space correction components, surgical planning and robotic control software, optical tracer systems, main consoles, and robotic arms. The intra-operative C-arm X-ray system was produced by Siemens, Germany. The cannulated screws were produced by Weigao Group, China (UC-pass pedicle screw system).

### Surgical methods

**Fig. 1:**
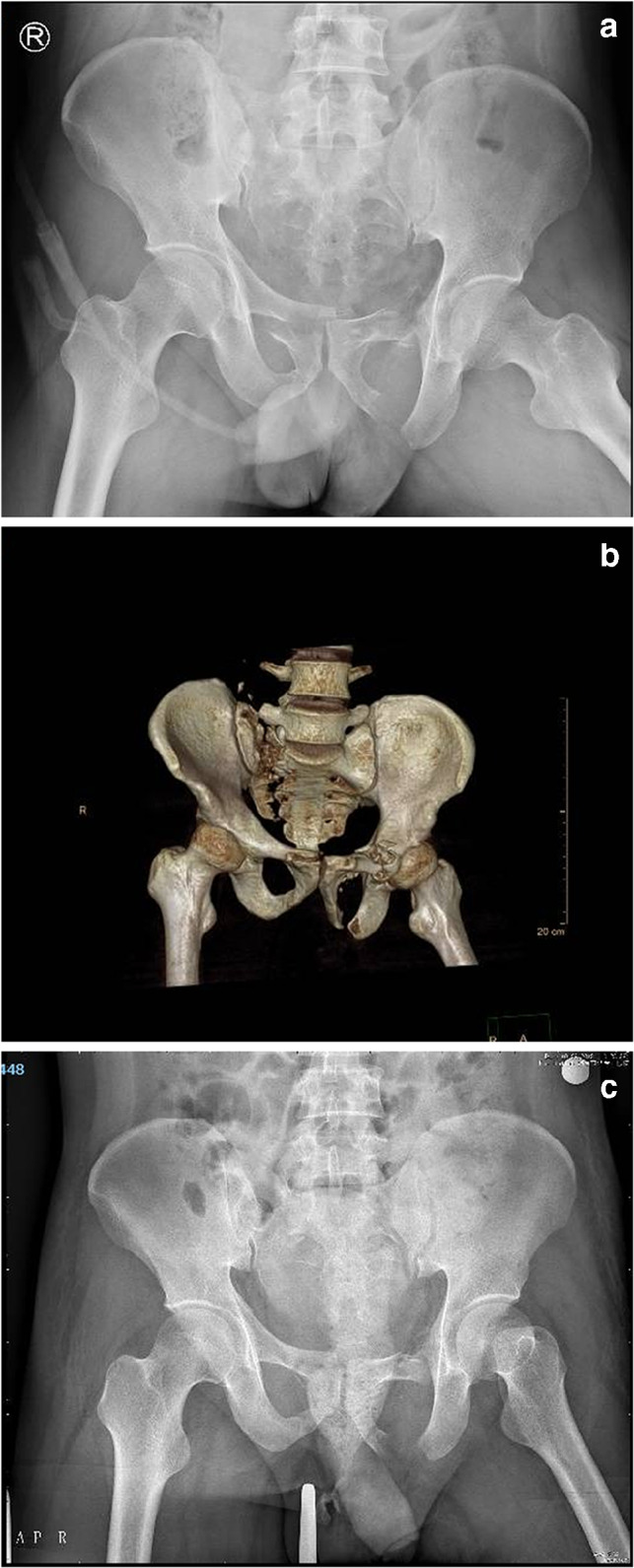
Example images from the cases of unstable pelvic fractures. **a** X-ray image of a male, 46 years old, with crush injury showing vertical displacement of the right sacroiliac joint, bilateral pubic ramus fracture, tile type C1. **b** 3D computed tomography (CT) reconstruction of a right sacral alar fracture, vertical displacement of the right sacroiliac joint and bilateral ramus pubis fracture. **c** Right lower limb bone traction, X-ray shows: right sacroiliac joint vertical displacement correction reduction, pelvic anterior and posterior ring reduction

**Fig. 2:**
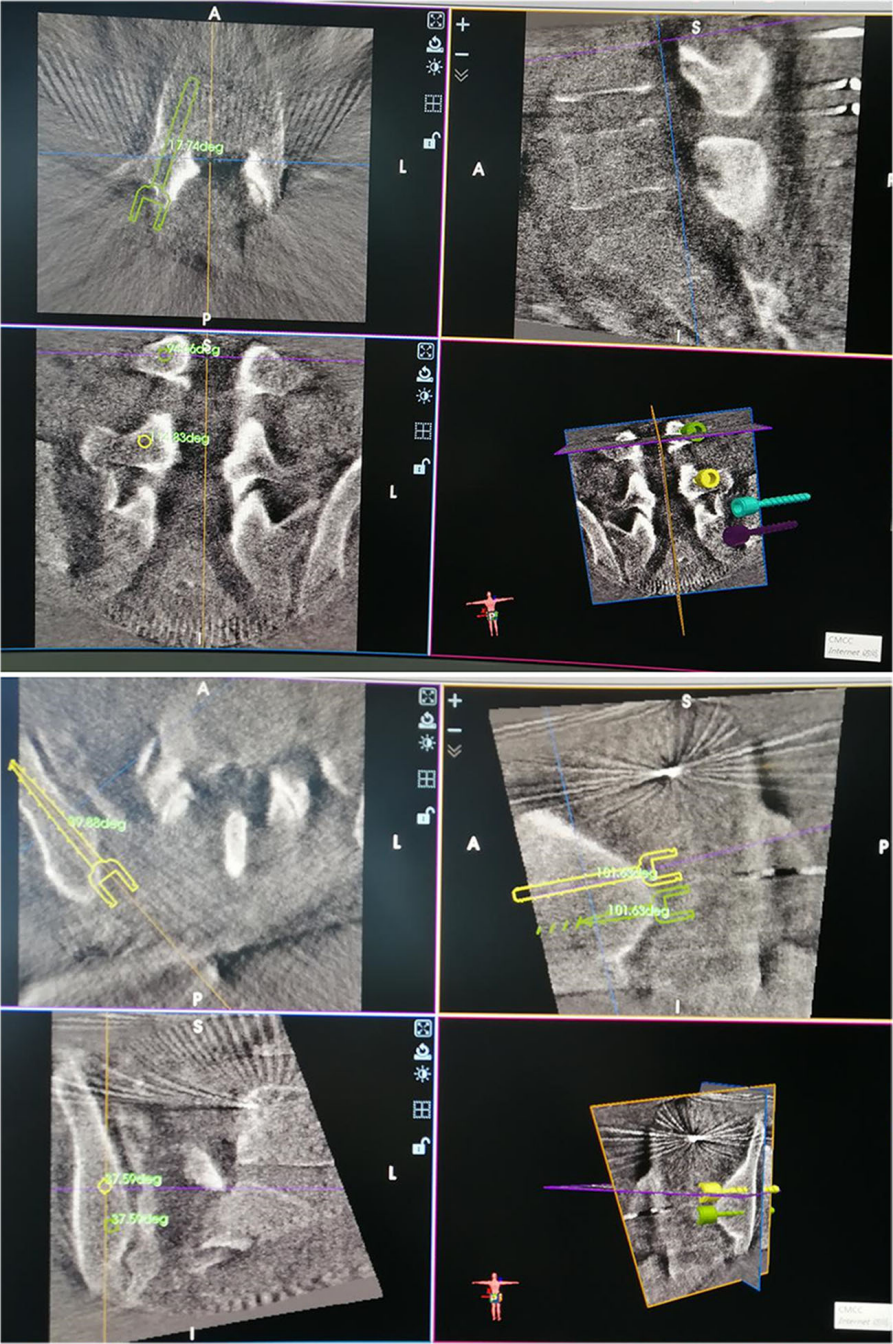
Images from the robot-assisted planning of the L4 and L5 pedicle screws, and iliac screw pathways

**Fig. 3:**
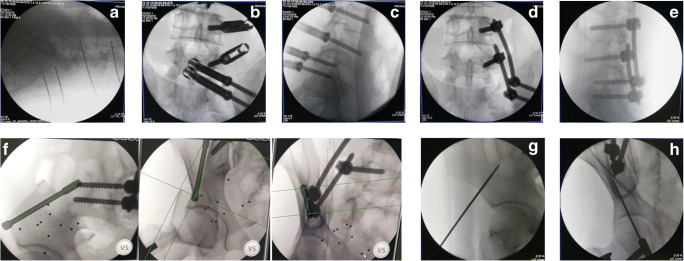
Robot-assisted percutaneous repair of the posterior ring with an iliac lumbar double rod fixator **a** pin placement assisted by robot guidance. **b**, **c** Cannulated pedicle screws were screwed according to the guide wire L4 and L5 into the right pedicle and the right ilium. **d**, **e** Under the appropriate length of titanium rod selection, the L4 pedicle screw end was connected with the cephalic iliac screw end, the L5 pedicle screw end was connected with the caudal iliac screw end, and the screw nut was tightened for fixation. **f** Anterior ring iliac cannulated screw nailing canal was planned according to anatomical landmarks at lacrimal position and iliac crest oblique position, the direction and specification of cannulated screw were insured. **g**, **h** A guide wire was inserted into the iliac plate at the anterior inferior iliac spine assisted by robot

All patients in this study were treated by the same orthopaedic surgeon, and the posterior iliac lumbar fixation was performed by the same spinal surgeon (deputy chief surgeon, with an annual operation rate of more than 500). The anterior ring INFIX fixation was performed by the same trauma surgeon (with an annual operation rate as chief surgeon of more than 800). The patient was intubated under general anaesthesia, prone on a Jackson spinal table and had traction boots on both feet. X-ray observation of pelvic reduction was in the prone position (a position change might cause a reduction of the pelvic displacement). If there was displacement, traction of the lower limb was performed with traction boots to reduce the posterior pelvic ring. The surgical field was disinfected, and a towel was placed. A 2-cm skin incision was made at the L3 spinous process, and subperiosteal stripping was performed to expose the L3 spinous process. A human tracer spinous process clip was then used to fix the L3 spinous process. The orthopaedic surgery robot was isolated with a sterile protective sleeve, a tracer and a positioning scale were installed at the end of the robot, and the positioning scale was placed in the operating field (with the sacroiliac joint on the injured side at the centre) (Fig. 1). The visual field with fluoroscopy included the injured side L4 and L5 vertebral pedicle and sacroiliac joint, and posterior iliac bone of the injured side. An intra-operative CT scan was performed, and the image was transmitted to the master console; the injured side L4 and L5 pedicle screw trajectory and iliac screw trajectory (Fig. 2) were planned. This ensured the correct placement direction and specification of the pedicle screws and iliac screws. Based on surgical planning, the robot automatically adjusted the posture and the end position of the mechanical arm. After the robot stopped running, the working sleeve was inserted along the robot end guided, a position which should ensure that the front end of the working sleeve touches the bone. The guide wire was inserted along the direction of the working sleeve (Fig. 3a), the skin was cut 2 cm along the guide wire, the muscle was expanded step by step, the screw was tapped, and the cannulated pedicle screw and iliac screw were screwed along the guide wire (Fig. 3b, c). Before iliac screw placement, iliac bone osteotomy was performed along the iliac screw guide needle, with an osteotomy depth of about 1 cm; this should ensure that the iliac screw tail was lower than the iliac bone after the tail fracture, and reduce the skin irritation caused by the tail nail after the surgery. The iliac screw depth was monitored by X-ray of the lateral view of the L4 and the L5 pedicle screw tails and iliac screw tail were approximately at the same height, conducive to percutaneous rod penetration and fixation. After pedicle screw placement was completed, an appropriate length titanium rod was selected, and the L4 pedicle screw end was connected with the cephalic iliac screw end through the percutaneous rod. The L5 pedicle end screw was connected with the caudal iliac screw end, and the screw was tightened and fixed (Fig. 3d, e). The ring was fixed when washing and suturing were finished.

The patient was then changed to the supine position, and robot-assisted percutaneous placement of cannulated pedicle screws in the anterior inferior iliac spine combined with INFIX fixator hollow screws (Weigao Group, China, UC-pass pedicle screw system) was performed. According to the anatomical markers, an anterior iliac screw trajectory was planned at the position of teardrops and the iliac oblique position, and the direction and specification of the cannulated screws were determined. Similarly, one cannulated 6.5 mm × 60 mm pedicle screw was inserted into the iliac plate at the bilateral anterior inferior iliac spine assisted by the robot (Fig. 3f–h). According to the shape of the patient’s abdomen, the connecting rod was pre-bent and then penetrated subcutaneously to connect the ends of the pedicle screws on both sides. According to the reduction of the anterior ring, the connecting rod could be pressed to the inner side for fixation, and the screw cap was tightened to complete the fixation of the anterior ring.

### Post-operative treatment

**Fig. 4:**
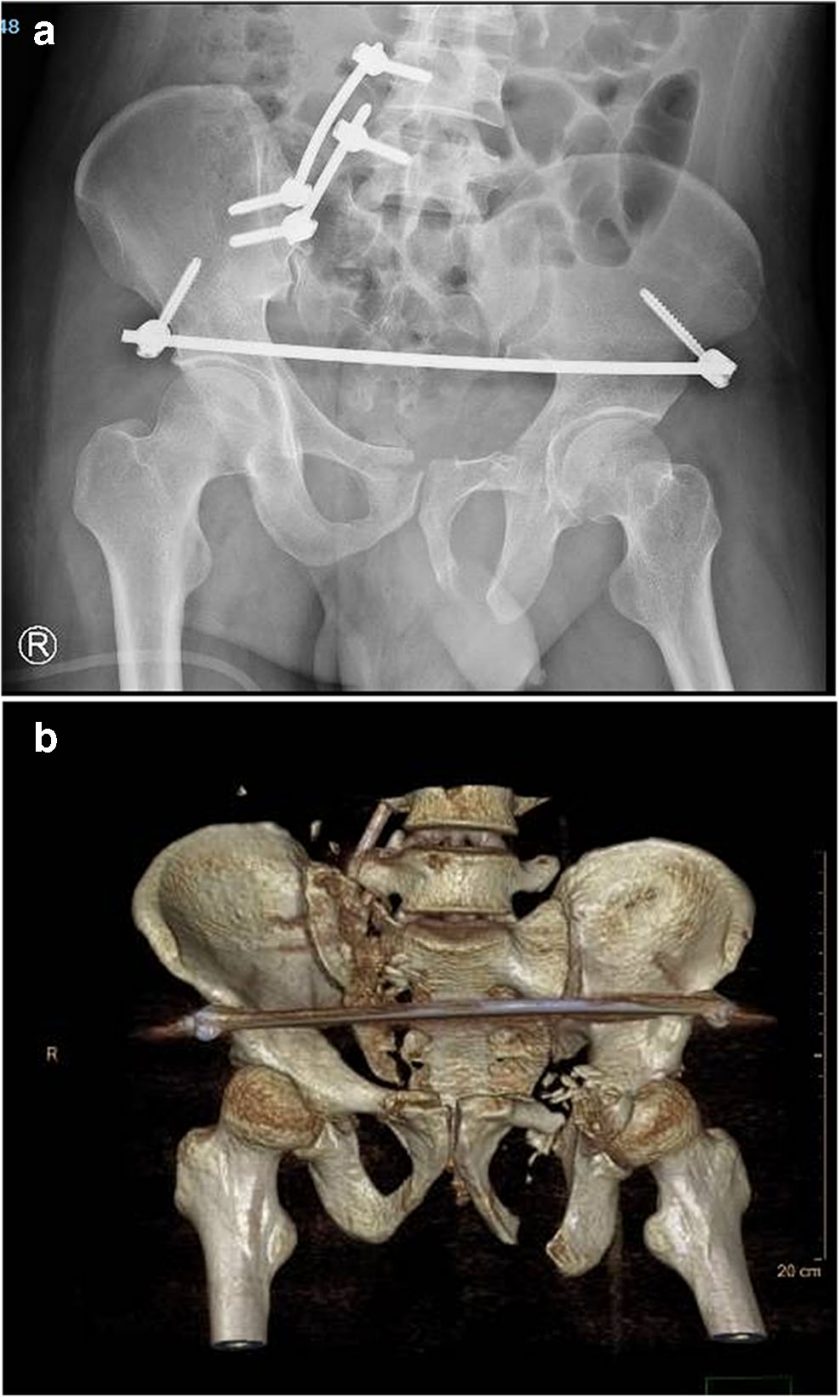
Post-operative images of the unstable pelvic fracture repair. **a** Post-operative X-ray showing vertical displacement reduction of the right sacroiliac joint at the posterior pelvic ring, reduction and good fixation of the pubic ramus fracture at the anterior pelvic ring. **b** Post-operative computed tomography (CT) 3D reconstruction showing good pelvic ring reduction and fixation

Post-operative infection prevention was provided after 48 hour using antibiotics. Prevention of lower extremity deep vein thrombosis (DVT) was provided for four  weeks by subcutaneous injection of nadroparin calcium injection (low molecular weight heparin, LMWH) 38 IU/kg at 12 hours (h) before surgery, 12 hours after surgery, and 24 hours after surgery, respectively. Afterwards, it was used once a day for three consecutive days. The dose was adjusted to 57 IU/kg from the fourth day after surgery until discharge. Within three hours of surgery, all patients accepted B-ultrasound examination of the lower limb vein to exclude DVT and accepted the use of an intermittent pneumatic compression device twice a day, each time for 60 minutes (min) until discharge. Finally, the patient was instructed to exercise the active and passive contractile functions of the lower extremity muscles. Pelvic orthography (Fig. 4a), outlet, entrance, lateral, CT, and three-dimensional reconstruction (Fig. 4b) were re-examined 72 hours after surgery. One week after the operation, the patient was advised to rest in bed, with turning over activities. During this period, the patient should undertake active and passive lower limb exercise and flexion and extension of the hip and knee joints.

### Data collection

The operation time and intra-operative bleeding were recorded. The degree of pelvic fracture displacement was recorded according to pre-operative and post-operative X-ray. The reduction effect of pelvic fracture displacement was evaluated according to Matta score [20], among which 4 mm was excellent, 4~10 mm was good, 10~20 mm was acceptable and > 20 mm was poor. Post-operative functional results were evaluated according to Majeed score [21]. A full score of 100~85 was considered excellent, 70~84 was good, 55~69 was acceptable, and 54 was poor. A pelvic X-ray was performed at one  month, three  months, six  months, and 12 months after surgery to evaluate screw position and pelvic fracture healing. The outpatient department reviewed the pelvic X-ray to evaluate the screw position and the healing of pelvic fractures, which included pelvic pain, lumbosacral pain, post-operative lumbar range of motion, as well as internal fixation loosening, fracture, pressure sores, and other complications. The results were analyzed based on the assessments six  months after surgery.

### Statistical analysis

Data analysis was performed using SPSS version 22.0 (IBM SPSS, IBM Corp., USA) and was presented as mean ± standard deviation for normally distributed data or median and range for non-normal distributed data.

## Results

### Baseline characteristics

In total, 17 cases of unstable fractures of the pelvis before and after closed ring (Fig. 1a, b), and patients with pelvic displacement were reduced by closed traction (Fig. 1c). The characteristics of the patients are shown in Table 1. All patients were Han Chinese, including 12 males and five females (age 21–71 years, 40.1 ± 3.8 years. The causes of injury were seven cases of fall injury, seven cases of traffic injury, and three cases of crush injury. Among these patients, four cases had combined rib fractures, three cases had thoracic and lumbar fractures, and two cases had cystorrhexis. Tile typing was B1 in seven cases, B2 in two cases, and C1 in eight cases.

**Table 1:** Clinical data of the patients treated for unstable pelvic fractures

Case number	Age (years)	Gender	Injury method	Injury to surgery time (days)	Tile type	Combined injury	Operation time (min)	Blood loss (mL)	Post-operative imaging Matta evaluation	Last follow-up Majeed score
1	50	Male	Falling	5	C1	Lumbar vertebra fracture	90	80	Excellent	88
2	21	Male	Traffic accident	6	C1	No	110	100	Excellent	98
3	34	Female	Falling	5	C1	No	120	120	Excellent	95
4	46	Male	Crush	14	C1	Cystorrhexis	120	110	Good	87
5	71	Male	Falling	7	B1	Rib fracture	120	90	Excellent	90
6	38	Male	Traffic accident	6	B1	Rib fracture	90	120	Excellent	88
7	26	Female	Traffic accident	5	B1	No	110	90	Excellent	96
8	38	Male	Crush	12	B2	Cystorrhexis	160	140	Good	85
9	65	Male	Traffic accident	5	B2	Thoracic fracture	120	100	Excellent	89
10	40	Female	Crush	4	B1	No	140	130	Excellent	93
11	48	Male	Falling	8	C1	Rib fracture	100	100	Excellent	95
12	35	Male	Falling	7	C1	Rib fracture	120	80	Excellent	92
13	27	Male	Traffic accident	5	B1	No	100	120	Excellent	96
14	24	Female	Falling	5	C1	No	110	80	Excellent	95
15	37	Male	Falling	6	C1	Lumbar vertebra fracture	110	90	Excellent	87
16	53	Female	Traffic accident	6	B1	No	120	100	Excellent	94
17	29	Male	Traffic accident	5	B1	No	100	150	Excellent	93

### Surgery

The operation time was 112.9 ± 16.8 minutes, and the blood loss was 105.9 ± 20.6 mL. A total of 34 lumbar pedicle screws and 34 iliac crest screws were placed.

### Follow-up

The mean follow-up time was 6.7 months (3–12 months). Post-operative CT scans showed that all the screws were well positioned in the medullary cavity. Post-operative imaging evaluated by Matta was excellent in 15 cases and good in two cases. The Majeed score in the last post-operative follow-up was 91.8 ± 3.8, and all 17 cases were excellent. A free thrombus of the lower extremity femoral vein occurred in two patients four  days after surgery and seven  days after surgery, and the thrombus disappeared aftertwo  weeks of anticoagulant treatment, and the filter was removed. One case had poor wound healing due to liquefaction of fat, which healed three  weeks after surgery after intensive dressing change and physical therapy with local light heating at the edge of the incision.

## Discussion

Unstable pelvic fractures usually involve injuries to the anterior and posterior pelvic rings. Ward et al. [22] suggested both anterior and posterior ring internal fixators were needed to stabilize the pelvic ring. For complicated pelvic anterior and posterior ring injuries, a combined approach before and after surgery will lead to huge surgical trauma and changing post-operative position might increase the possibility of wound infection, which is bad for early post-operative rehabilitation exercise. In our clinic, we designed a robot-assisted percutaneous iliac lumbar fixator combined with INFIX to treat these fractures. The results of this case series showed the operation time was 112.9 ± 16.8 minutes, and bleeding was 105.9 ± 20.6 mL. Matta evaluation was excellent in 15 and good in two cases. Majeed score at last follow-up was 85–98 points, excellent in all 17 cases. There was a low rate of complications; two cases of lower extremity deep vein thrombosis received an inferior vena cava filter. One case showed incision fat liquefaction healing. These results suggest this innovative method provided perfect pelvic stability, short surgical time; and bleeding with few complications related to the iliopsoas fixator.

The results of this study compare well to previous studies with other methods of treating unstable pelvic fractures. A comparison of sacroiliac anterior plate fixation (SAPF), sacroiliac anterior papilionaceous plate (SAPP), and percutaneous sacroiliac screw internal fixation (PSCIF) showed the operation time for SAPF was 118.5  ±  20.6  minutes, PSCIF was 88.8  ±  14.0  minutes, and SAPP was 106.6  ±  17.2  minutes. Blood loss for SAPF was 653.8  ±  144.5 mL, SAPP 570.8  ±  127.5 mL, and PSCIF 88.8 ± 14.0 mL. The Matta score was excellent: 12, good: five, acceptable: four, poor: five for SAPF; excellent: 15, good: eight, acceptable: three, poor: zero for SAPP; and excellent: 16, good: nine, acceptable: one, poor for PSCIF. The Majeed score was excellent: 14, good: ten, acceptable: two, poor: zero for PSCIF; excellent: six, good: 11, acceptable: eight, poor: one for SAPF; and excellent: 13, good: nine, acceptable: four, poor: zaero for SAPP [23]. Another study of 22 cases showed that treatment with plate fixation of the anterior ring with the Stoppa approach classified the quality of reduction by Matta method as 16 anatomical and six nearly anatomical reductions. The functional results were classified as seven excellent, 12 good, and three fair by Merle d’Aubigne-Postel score [24]. A method that aimed to be less invasive ilioinguinal approach combined with a minimally invasive posterior approach in 37 patients showed anatomical or near to anatomical reduction in [25] anterior pelvic ring fractures and a satisfactory result in 11. For the posterior sacral fractures, excellent reduction was obtained in 33 but a residual deformity in 4 patients. The clinical outcome by Majeed score at one  year was “excellent” in 29 patients and “good” in eight patients [25].

**Fig. 5:**
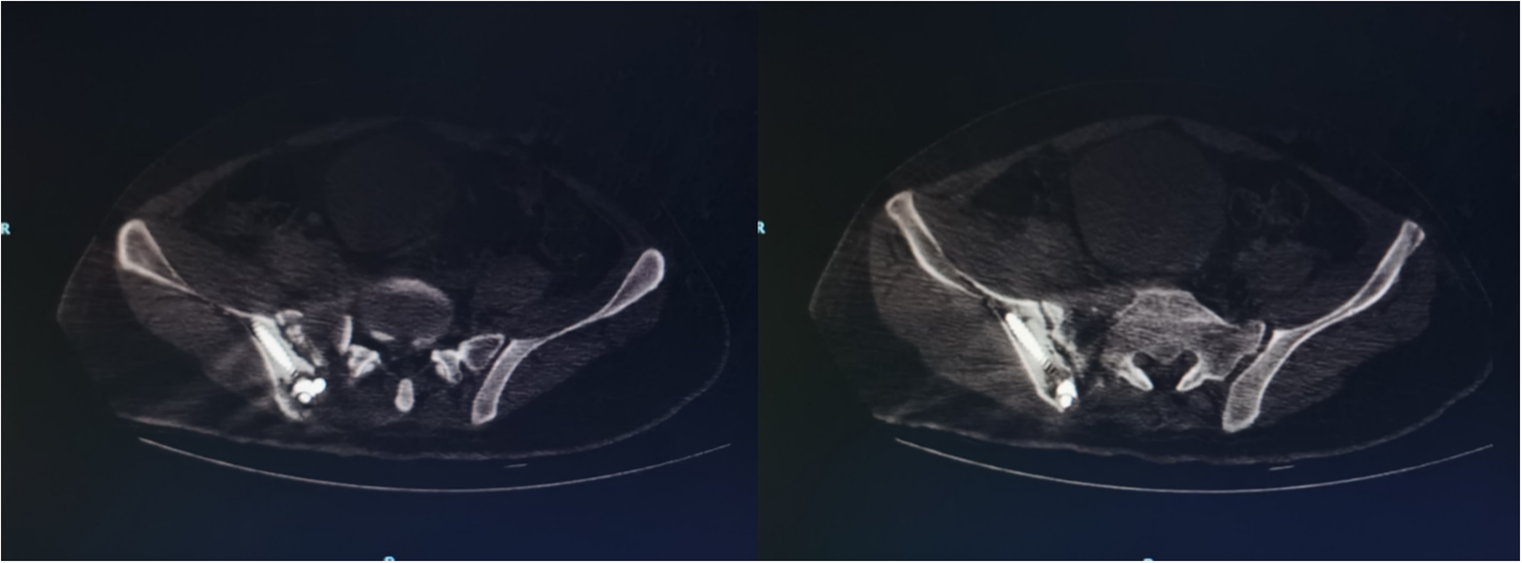
CT scan showed that the iliac screws was in good position and did not puncture the iliac cortex

The lumbosacral junction is a stress concentration site that transfers upper-body weight to the iliac lower limbs through the sacrum, injury in this position seriously affects the conduction of axial stress to the pelvis and lower limbs, and a strong internal fixator is required to maintain the reconstruction and stability of the spine and pelvis. Iliac screw reconstruction of the lumbosacral junction has shown superior biomechanical characteristics to other internal fixator methods [7]. Therefore, we used an improved minimally invasive technique with robot assistance to link the L4 pedicle screw end with the cephalic iliac screw end and link the L5 pedicle screw end with the caudal iliac screw end. The iliac screw trajectories should be parallel with L4 and L5 pedicle screws, and the tail heights of iliac screws and pedicle screws should be as same as possible on lateral fluoroscopic images; otherwise, the U type mouth of screws do not attach with titanium rods and the nut will not be tighten with the rod and will be out from the U type mouth of screws. Based on the considerations, during the intra-operative planning, we measured the iliac screw about 50 mm avoiding to wear out iliac lamella. So the length of the double iliac screws was relatively short (Fig. 5).

**Fig. 6:**
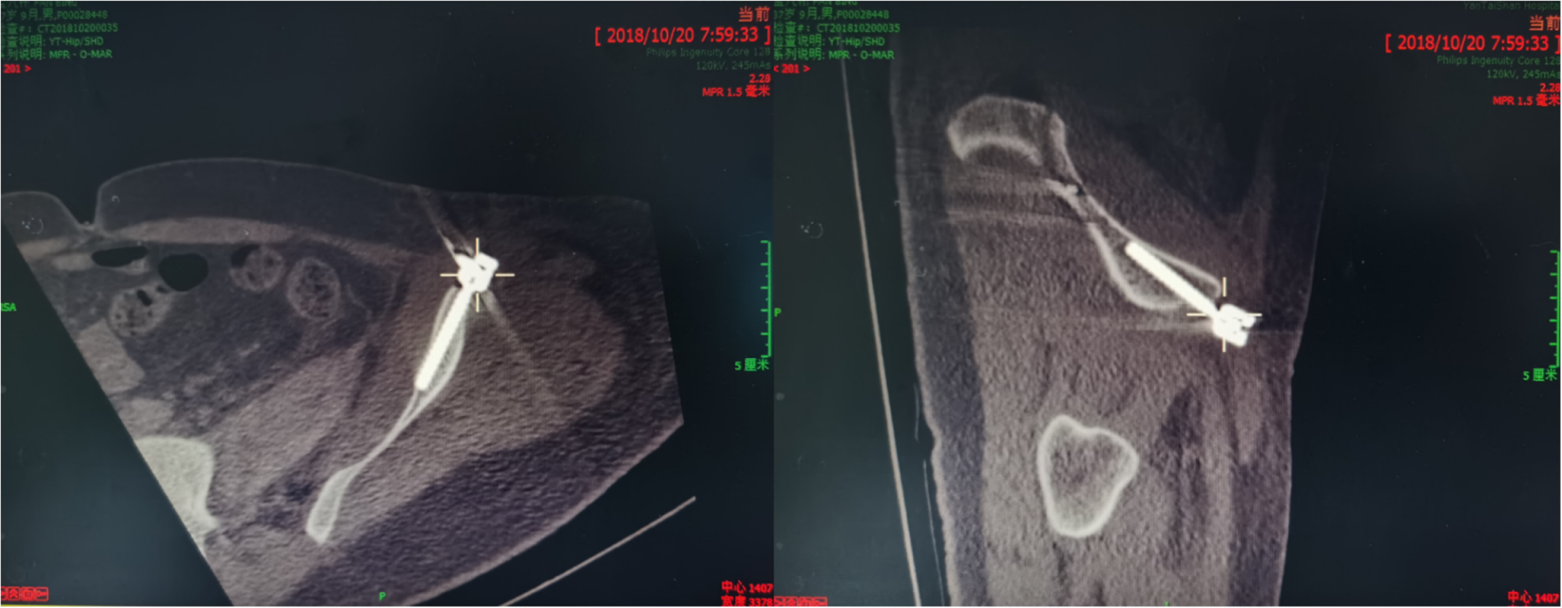
Post-operative CT scan reconstruction of the INFIX screws showed that the iliac cortex could be perforated if a longer screw was selected

**Fig. 7:**
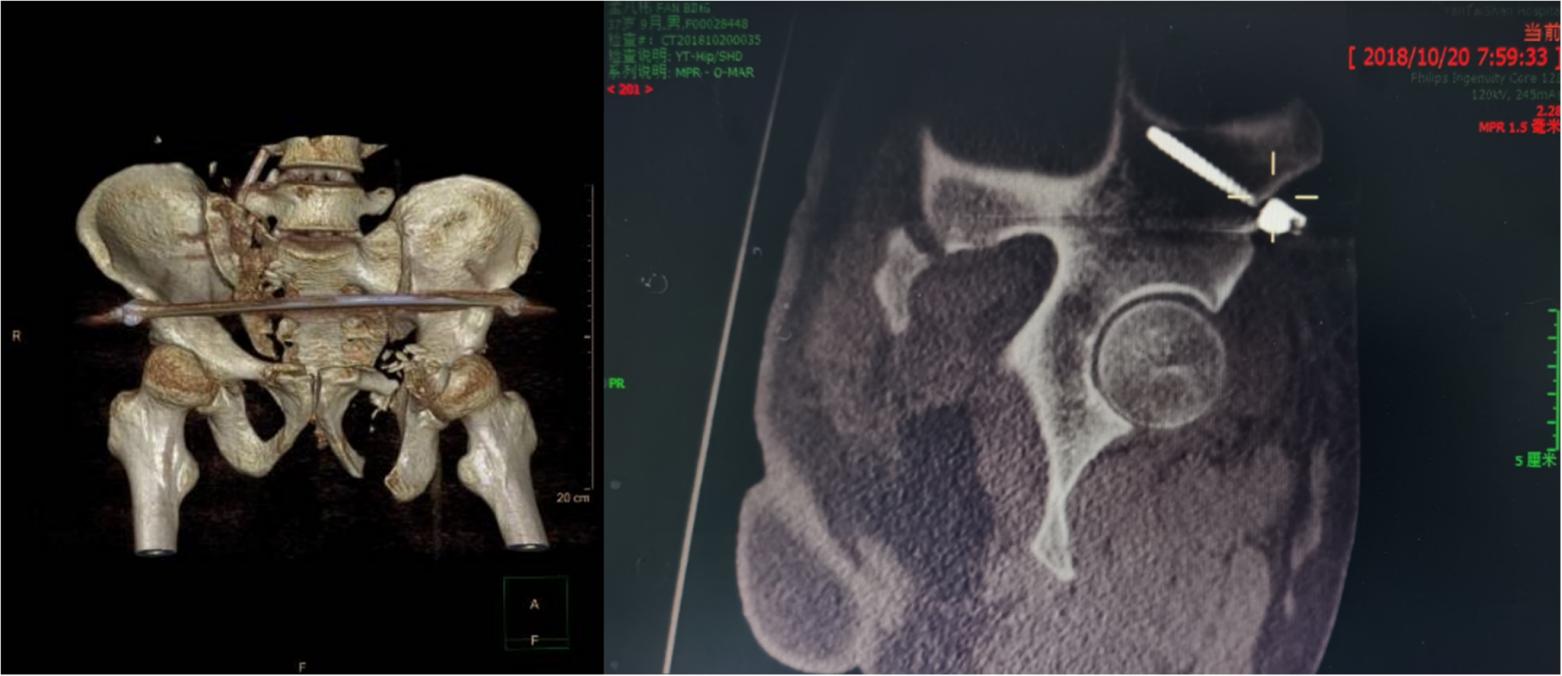
From the three-dimensional reconstruction and the screw reconstruction, the position of the starting point of the left anterior inferior iliac screw is correct

For the anterior ring, Vaidya [18, 26] suggested INFIX indications included superior ramus pubis, inferior ramus pubis, and bilateral ramus pubis fractures. All 17 patients in this group received an INFIX fixator and one successful screw placement. The Ti-robot guided surgeons making the method easy, effective, and safe to complete according to pre-operative planning. According to the anterior inferior iliac spine, an anterior iliac ring cannulated screw canal was planned at the position of teardrops and the iliac oblique position, and the direction of the cannulated screws were determined. However, the X-rays could not determine the length of the INFIX screws, and we were concerned that the screws might puncture the medial and lateral cortex, so we chose a 6.5 mm × 60 mm pedicle screw. Postoperative CT scan reconstruction of the INFIX screws showed that the medial and lateral cortex could be perforated if a longer screw was selected (Fig. 6). From the three-dimensional reconstruction and the screw reconstruction, the position of the starting point of the left anterior inferior iliac screw is correct (Fig. 7). According to post-operative X-rays and CT scan, the left SI joint of the patient had disruption, but the position was good. The displacement of the right sacroiliac joint and the fracture of the upper and lower ramus of the left pubis were the main reasons for the instability of the pelvic ring, and the left SI joint disruption was not the main reason for the instability of the pelvic ring, so we did not fix it.

The procedure had minor trauma, with no lateral femoral cutaneous nerve injury, infection, failure of the internal fixator, and other complications that occurred. The patients’ daily activities were unaffected, and they felt comfortable.

We suggest some surgical indications and precautions for the use of this method in patients with unstable pelvic fracture. The closed pelvic anterior and posterior ring injuries should not have caused severe internal organ injuries, with no obvious fracture displacement, or displacement by pre-operative and intra-operative traction. Patients with severe internal organ injuries such as bladder rupture, urethral rupture, and intestinal rupture require emergency laparotomy, and when obvious wound contamination has occurred, the patients were contraindicated for surgery.

The suggested surgical order is the posterior ring fixator and then the anterior ring fixator. For patients with vertical instability, high-weight bone traction was used to correct vertical displacement of fractures.

In this study, robot-assisted treatment with a transcutaneous iliac lumbar fixation combined with an INFIX fixation showed a favorable effect in the treatment of unstable pelvic fractures. We consider this method has some advantages over other methods. The robot provided favorable iliac screw and INFIX screw access: no screws penetrated the cortex. The robot shortened the operation time and improved operation efficiency. In this study, it is important to note that the operation time included the time for surgery and CT scan, and included intra-operative process planning and intra-operative optical tracer with optical tracking technology, which required repeated perspective and accurate positioning. Due to the robot’s precise positioning, the fixed pedicle iliac waist and iliac screws nail punctures were successful for the first time and this reduced the operation time. Compared with using a freehand screw fixation, robot-assisted surgery had significantly fewer intra-operative fluoroscopy times; thus, it reduced the cumulative intra-operative radiation dose.

This study also has some limitations. This technique was developed in a short time and the number of patients who underwent this procedure is small and from one hospital. There was no control group to compare the outcomes with a more traditional technique. More cases from multiple centers and prospective, randomized controlled studies with long-term follow-up are needed to fully demonstrate its benefits.

Robot-assisted treatment of unstable pelvic fractures with a percutaneous iliac lumbar double bar fixation combined with a percutaneous anterior pelvic ring INFIX fixation was a feasible choice and minimally invasive. Therefore, this method should be considered an option for the treatment of unstable pelvic fractures.
